# Multiplicative noise removal using primal–dual and reweighted alternating minimization

**DOI:** 10.1186/s40064-016-1807-3

**Published:** 2016-03-05

**Authors:** Xudong Wang, Yingzhou Bi, Xiangchu Feng, Leigang Huo

**Affiliations:** School of Computer and Information Engineering, Guangxi Teachers Education University, Nanning, 530001 China; School of Mathematics and Statistics, Xidian University, Xi’an, 710126 China

**Keywords:** Image denoising, Multiplicative noise, Artificial parameter, Primal–dual algorithm

## Abstract

Multiplicative noise removal is an important research topic in image processing field. An algorithm using reweighted alternating minimization to remove this kind of noise is proposed in our preliminary work. While achieving good results, a small parameter is needed to avoid the denominator vanishing. We find that the parameter has important influence on numerical results and has to be chosen carefully. In this paper a primal–dual algorithm is designed without the artificial parameter. Numerical experiments show that the new algorithm can get a good visual quality, overcome staircase effects and preserve the edges, while maintaining high signal-to-noise ratio.

Multiplicative noise appears in many image processing applications, such as synthetic aperture radar (SAR), ultrasound imaging, single particle emission-computed tomography, and positron emission tomography. It reduces the image quality seriously and affected the subsequent processing, The traditional Gauss-based distribution denoising models (Rudin et al. 1992; Wu and Tai 2010) are not suitable for removing this sort of noise. Hence the construction of multiplicative noise model and the corresponding efficient algorithm become an important research topic recently.

Gamma distribution is commonly used to simulate multiplicative noise. Based on this assumption many models have been established. Aubert and Aujol (2008) put forward AA model using maximum a posteriori probability (MAP). Based on the logarithm transform, Shi and Osher (2008) proposed SO model. Huang et al. (2009) presented linearized alternating direction methods HNW, and Chen and Zhou (2014) proposed a linearized alternating direction method using discrepancy function constraint (Huang et al. 2013). In order to better protect the edge of the denoised image, Wang et al. (2012) suggested an iteratively reweighted total variation model (IR-TV). In this model the expectation maximum (EM) and the total variation (TV) with the classical iteratively reweighted algorithm are used. In order to avoid zero denominator in the iterative process, an artificial parameter is needed. It is well known that the parameter has important influence on numerical results and has to be chosen carefully. In this paper, an improvement of the iteratively reweighted algorithm is introduced without the artificial parameter,

The rest of the paper is organized as follows: “Iteratively reweighted model with TV” section briefly introduces the IR-TV model as well as the classical iteratively reweighted algorithm. “Solution to the model” section presents the new algorithm of IR-TV model, which is based on the primal–dual optimization and without the artificial parameter. In “Numerical experiment” section the effectiveness of the proposed algorithm is verified through numerical experiments. Finally we conclude in “Conclusion” section.

## Iteratively reweighted model with TV

Suppose the degraded image $f({\mathbf{x}}) = u({\mathbf{x}})v({\mathbf{x}}),x \in\Omega$, where the original image $u({\mathbf{x}})$ is a real piecewise smooth function defined on a bounded domain $\Omega  \subset R^{2}$, and the multiplicative noise $v({\mathbf{x}})$ is assumed to obey Gamma distribution with mean 1

1 $$ {\text{g}}_{{\rm v}} (v) = \left\{ {\begin{array}{ll} {\frac{{L^{L} v^{L - 1} }}{\varGamma \left( L \right)}e^{ - Lv} ,} &\quad {v > 0} \\ {0,} & \quad{v \le 0} \\ \end{array} } \right. $$

In Eq. (1), $\varGamma ( \cdot )$ is a Gamma function with variance 1/*L*.

Iteratively reweighted *l*_1_ regularization minimization problem attempts to find a local minimum of concave penalty functions that more closely resembles the *l*_0_ regularization problem (Simon and Lai 2009; Candes et al. 2008). In our previous work Wang et al. (2012), we put forward an iteratively reweighted model

2 $$ z_{op} = \arg \mathop {\hbox{min} }\limits_{z} \left\{ {\mu \int_{\Omega } {g({\mathbf{x}})\phi \left( z \right)dx_{1} dx_{2} } } \right. + \left. {\int_{\Omega } {\left( {z + fe^{ - z} } \right)dx_{1} dx_{2} } } \right\} $$

where $z({\mathbf{x}}) = \log u({\mathbf{x}})$ and $\phi (z) = |\nabla z|$ ,regularizer parameter *μ* is a constant connected with the intensity of noise, $g({\mathbf{x}})$ is a nonnegative weight function which controls the strength of smoothing. According to the classical iteratively reweighted algorithm, we choose

3 $$ g\left( {\mathbf{x}} \right) = \left\{ \begin{aligned} 1\quad \;\quad \quad \;\quad \quad \;\;{\kern 1pt} n = 1 \hfill \\ \frac{1}{{\left| {\nabla z^{{\left( {n - 1} \right)}} \left( {\mathbf{x}} \right)} \right|}}\quad \;\quad n \ge 2 \hfill \\ \end{aligned} \right. $$

where *n* is the number of outer iteration. It is obvious that the larger $\left| {\nabla z} \right|$, the weaker smoothing strength is, thus the noise is removed while the edges are preserved.

The classical algorithm to Eq. (2) attempts to find a local minimum of a concave function, whereas in each iteration the algorithm simply requires to solve a convex optimization problem, In order to prevent the zero denominator, Eq. (3) usually be revised to

4 $$ g\left( {\mathbf{x}} \right) = \left\{ \begin{aligned} 1\quad \;\quad \quad \;\quad \quad \quad \quad \,\;\;{\kern 1pt} n = 1 \hfill \\ \frac{1}{{\left| {\nabla z^{{\left( {n - 1} \right)}} \left( {\mathbf{x}} \right)} \right| + \varepsilon^{\left( n \right)} }}\quad \;\quad n \ge 2 \hfill \\ \end{aligned} \right. $$

The parameter $\varepsilon^{\left( n \right)}$ provides the stability for iterations. The choice of $\varepsilon^{\left( n \right)}$ has a significant effect on the result of the denoising. Therefore it needs to carefully adjusted. It will lead to poor denoising results with a inappropriate $\varepsilon^{\left( n \right)}$ (Wang et al. 2012; Simon and Lai 2009).

In next section, we propose a novel algorithm to solve Eq. (2). First the splitting method is used to transform the original equation into two corresponding equations. Then the primal–dual algorithm and the Euler–Lagrange method are applied to solve these two subproblems respectively.

## Solution to the model

As Huang et al. (2009) has mentioned, let us consider the splitting form of Eq. (2)

5 $$ \mathop {\hbox{min} }\limits_{w,z} J\left( {w,z} \right) = \mathop {\hbox{min} }\limits_{w,z} \left\{ {\int_{\Omega } {\left( {z + fe^{ - z} } \right)dx_{1} dx_{2} } + \gamma \int_{\Omega } {\left( {w - z} \right)^{2} dx_{1} dx_{2} } + \mu \int_{\Omega } {g\left( {\mathbf{x}} \right)\left| {\nabla w} \right|dx_{1} dx_{2} } } \right\} $$

where *w* is an auxiliary function, The parameter *γ* measures the amount of regularization to a denoising image, which is large enough to make *w* be close to *z*. In our experiment, *γ* = 19 is chosen. The main advantage of the proposed method is that the TV norm can be used in the noise removal process in an efficient manner. And Eq. (5) can be splitted into two equations

6a $$ w^{\left( n \right)} = \arg \mathop {\hbox{min} }\limits_{w} \left\{ {\gamma \int_{\Omega } {\left( {w - z^{{\left( {n - 1} \right)}} } \right)^{2} dx_{1} dx_{2} } + \mu \int_{\Omega } {g\left( {\mathbf{x}} \right)\left| {\nabla w} \right|dx_{1} dx_{2} } } \right\} $$

6b $$ z^{\left( n \right)} = \arg \mathop {\hbox{min} }\limits_{z} \left\{ {\int_{\Omega } {\left( {z + fe^{ - z} } \right)dx_{1} dx_{2} } + \gamma \int_{\Omega } {\left( {w^{\left( n \right)} - z} \right)^{2} dx_{1} dx_{2} } } \right\} $$

This is an alternating minimization algorithm. The first step of the method is to apply a weighted TV denoising scheme to the image generated by the previous multiplicative noise removal step. The second step of the method is to solve a part of the optimization problem.

In this paper, a primal–dual algorithm (Bertsekas et al. 2006; Bertsekas 2011) is applied to iteratively reweighted model 6a. Convex close set *K* is defined by,

$$ K = \overline{{\left\{ {divp|p \in C_{c}^{1} \left( {\Omega ,{\mathbb{R}}^{2} } \right),\left| p \right| \le g\left( {\mathbf{x}} \right),\forall {\mathbf{x}} \in \Omega } \right\}}} $$

where $\overline{{\{ \cdot \} }}$ denotes convex close set of $\{ \cdot \}$.

Let $X,Y$ be two finite dimention real vector spaces, the corresponding norm defined as $\left\| \cdot \right\| = \left\langle { \cdot , \cdot } \right\rangle^{1/2}$, where $\left\langle { \cdot , \cdot } \right\rangle$ is the inner product. Gradient operator $\nabla :X \to Y$ is continuous linear operator, the corresponding norm defined as

$$ \left\| \nabla \right\| = \hbox{max} \left\{ {\left\| {\nabla {\mathbf{x}}} \right\|\;|\;\left\| {\mathbf{x}} \right\| \le 1,{\mathbf{x}} \in X} \right\} $$

We introduce a divergence operator $div:X \to Y$, the adjoint of divergence operator is defined by $\nabla^{ * } = - div$. Then we introduce dual variable $p = \left( {p_{1} ,\,p_{2} } \right)$, which divergence is $divp = {{\partial p_{1} } \mathord{\left/ {\vphantom {{\partial p_{1} } {\partial x_{1} }}} \right. \kern-0pt} {\partial x_{1} }} + {{\partial p_{2} } \mathord{\left/ {\vphantom {{\partial p_{2} } {\partial x_{2} }}} \right. \kern-0pt} {\partial x_{2} }}$, we have

$$ \left\langle {\nabla w,\;p} \right\rangle_{Y} = - \left\langle {w,\;divp} \right\rangle_{X} $$

The regularizator of 6a is

$$ J\left( w \right) = \int_{\Omega } {g\left( {\mathbf{x}} \right)\left| {\nabla w} \right|dx_{1} dx_{2} } = \mathop {\sup }\limits_{p} \left\{ {\int_{\Omega } {wdivpdx_{1} dx_{2} } \left| {p \in C_{c}^{1} \left( {\Omega ,{\mathbb{R}}^{2} } \right),\left| p \right| \le g\left( {\mathbf{x}} \right),\forall {\mathbf{x}} \in \Omega } \right.} \right\} $$

and 6a can be transformed into

7 $$ \mathop {\hbox{min} }\limits_{w} \left\{ {\frac{\gamma }{\mu }\int_{\Omega } {\left( {w - z^{{\left( {n - 1} \right)}} } \right)^{2} dx_{1} dx_{2} } + J\left( w \right)} \right\} $$

for every $w \in X$ and $\lambda > 0$, $J\left( {\lambda w} \right) = \lambda J\left( w \right)$ holds, so *J* is one-homogeneous. By the Legendre–Fenchel transform, we can obtain

$$ J^{ * } \left( v \right) = \mathop {\sup }\limits_{w} \left\langle {w,v} \right\rangle_{X} - J\left( w \right) $$

with $J^{ * } \left( v \right)$ is the “characteristic function” of a closed convex set *K*:

8 $$ J^{ * } \left( v \right) = \delta_{K} \left( v \right) = \left\{ {\begin{array}{ll} 0 &\quad {if\;v \in K} \\ { + \infty } &\quad {if\;v \notin K} \\ \end{array} } \right. $$

Since $J^{ * * } = J$, we recover

$$ J\left( w \right) = \mathop {\sup }\limits_{v \in K} \left\langle {w,v} \right\rangle_{X} $$

The Euler equation for (7) is

$$ 0 \in \frac{2\gamma }{\mu }\left( {w - z^{{\left( {n - 1} \right)}} } \right) + \partial J\left( w \right) $$

where $\partial J$ is the “sub-differential” of *J*. Writing this as

$$ \frac{2\gamma }{\mu }z^{{\left( {n - 1} \right)}} \in \frac{{2\gamma \left( {z^{{\left( {n - 1} \right)}} - w} \right)}}{\mu } + \frac{2\gamma }{\mu }\partial J\left( {\frac{{2\gamma \left( {z^{{\left( {n - 1} \right)}} - w} \right)}}{\mu }} \right) $$

we get that $q = 2\gamma \left( {z^{{\left( {n - 1} \right)}} - w} \right)/\mu$ is the minimizer of $\left\| {q - 2\gamma z^{{\left( {n - 1} \right)}} /\mu } \right\|^{2} + \frac{2\gamma }{\mu }J^{ * } \left( q \right)$. Since $J^{ * }$ is given by (3), the solution of problem (6) is simply given by

$$ w^{\left( n \right)} = z^{{\left( {n - 1} \right)}} - \pi_{{\frac{\mu }{2\gamma }K}} \left( {z^{{\left( {n - 1} \right)}} } \right) $$

Therefore the problem to compute $w^{\left( n \right)}$ become a problem to compute the nonlinear projection $q = \pi_{{{{\mu K} \mathord{\left/ {\vphantom {{\mu K} {2\gamma }}} \right. \kern-0pt} {2\gamma }}}} \left( {z^{{\left( {n - 1} \right)}} } \right)$. Consider the following problem:

9 $$ \mathop {{min} }\limits_{{p\left( {\mathbf{x}} \right) \in P}} \left\{ {\left\| {\frac{\mu }{2\gamma }divp - z^{{\left( {n - 1} \right)}} } \right\|^{2} |p \in C_{c}^{1} \left( {\Omega ,{\mathbb{R}}^{2} } \right),\left| p \right| \le g\left( {\mathbf{x}} \right),\forall {\mathbf{x}} \in \Omega } \right\} $$

Following the standard arguments in convex analysis (Chambolle 2004; Chambolle and Pock 2011), the Karush–Kuhn–Tucker conditions yield the existence of a Lagrange multiplier $\alpha_{i,j} \left( {\mathbf{x}} \right) \ge 0$, such that constraint problem (9) become to,

10 $$ - \left( {\nabla \left( {\frac{\mu }{2\gamma }divp - z^{{\left( {n - 1} \right)}} } \right)} \right)_{i,j} + \alpha_{i,j} \left( {\mathbf{x}} \right)p_{i,j} = 0 $$

Notice constraint problem $\left| p \right| \le g$ in Eq. (10). For any ${\mathbf{x}}$, $\alpha \left( {\mathbf{x}} \right) \ge 0$, if $\left| p \right|^{2} < g^{2}$ ,then $\alpha \left( {\mathbf{x}} \right) = 0$; If $\left| p \right|^{2} = g^{2}$, we see that in any case

$$ \left| {\nabla \left( {z^{{\left( {n - 1} \right)}} - \frac{\mu }{2\gamma }\left( {{\text{div}}p} \right)} \right)} \right|^{2} - \alpha^{2} \left( {\mathbf{x}} \right)g^{2} = 0 $$

Then

11 $$ \alpha \left( {\mathbf{x}} \right) = \frac{{\left| {\nabla \left( {z^{{\left( {n - 1} \right)}} - \frac{\mu }{2\gamma }\left( {{\text{div}}p} \right)} \right)} \right|}}{g} $$

Substituting (11) into (10) gives,

$$ \nabla \left( {z^{{\left( {n - 1} \right)}} - \frac{\mu }{2\gamma }\left( {{\text{div}}p} \right)} \right) + \frac{{\left| {\nabla \left( {z^{{\left( {n - 1} \right)}} - \frac{\mu }{2\gamma }\left( {{\text{div}}p} \right)} \right)} \right|}}{g}p = 0 $$

We thus propose the following semi-implicit gradient descent (or fixed point) algorithm. We choose τ > 0, let $p_{0} = 0$ and for any n ≥ 0,

12 $$ p_{m + 1} = \frac{{p_{m} + \delta t\nabla \left( {div\left( {p_{m} } \right) - \frac{2\gamma }{\mu }z^{{\left( {n - 1} \right)}} } \right)}}{{1 + \frac{\delta t}{{g\left( {\mathbf{x}} \right)}}\left| {\nabla \left( {div\left( {p_{m} } \right) - \frac{2\gamma }{\mu }z^{{\left( {n - 1} \right)}} } \right)} \right|}} $$

Combining Eq. (3) $$ g\left( {\mathbf{x}} \right) = \frac{1}{{\left| {\nabla z^{{\left( {n - 1} \right)}} } \right|}} $$, we calculate $$ p_{m + 1} $$$$ \left( {m \ge 1} \right) $$ by

13 $$ p_{m + 1} = \frac{{p_{m} + \delta t\nabla \left( {{\text{div}}\left( {p_{m} } \right) - \frac{2\gamma }{\mu }z^{{\left( {n - 1} \right)}} } \right)}}{{1 + \delta t\left| {\nabla z^{{\left( {n - 1} \right)}} } \right|\left| {\nabla \left( {{\text{div}}\left( {p_{m} } \right) - \frac{2\gamma }{\mu }z^{{\left( {n - 1} \right)}} } \right)} \right|}} $$

The denominator of Eq. (13) is greater than zero, which avoids the appearance of the rectified parameters, and of course does not need to be adjusted. The method can be seen a new method to solve nonconvex problem. We need to calculate the boundary of the norm $\left\| {div} \right\|$.

### **Theorem 1**

(Chambolle 2004) *If*$\kappa = \left\| \Delta \right\| = \left\| {div} \right\|$, *then*$\kappa^{2} \le 8$

Similar to Papers (Chambolle 2004; Chambolle and Pock 2011; Bresson et al. 2007), we now can show the following result about dual algorithm to iteratively reweighted TV model.

### **Theorem 2**

*Let*$\delta t \le {1 \mathord{\left/ {\vphantom {1 8}} \right. \kern-0pt} 8}$. *Then*, $\frac{\mu }{2\gamma }divp_{m}$*converges to*$\pi_{{{{\mu K} \mathord{\left/ {\vphantom {{\mu K} {2\gamma }}} \right. \kern-0pt} {2\gamma }}}} \left( {z^{{\left( {n - 1} \right)}} } \right)$*as*$m \to \infty$.

### *Proof*

By algorithm we easily see that for every $m \ge 0$, $\left| {\left( {p_{m} } \right)_{i,j} } \right| \le \left( {g\left( {\mathbf{x}} \right)} \right)_{i,j}$. Let $\eta = {{\left( {p_{m + 1} - p_{m} } \right)} \mathord{\left/ {\vphantom {{\left( {p_{m + 1} - p_{m} } \right)} \tau }} \right. \kern-0pt} \tau },$ it can be obtained

$$ \eta = \left( {\nabla \left( {z^{{\left( {n - 1} \right)}} - \frac{\mu }{2\gamma }\left( {{\text{div}}p_{m} } \right)} \right)} \right) + \frac{{\left| {\left( {\nabla \left( {z^{{\left( {n - 1} \right)}} - \frac{\mu }{2\gamma }\left( {{\text{div}}p_{m} } \right)} \right)} \right)} \right|}}{g}\left( {p_{m + 1} } \right), $$

Then we have

$$\begin{aligned} &\left\| {divp_{m + 1} - \frac{{2\gamma z^{{\left( {n - 1} \right)}} }}{\mu }} \right\|^{2} = \left\| {divp_{m} + \tau div\eta - \frac{{2\gamma z^{{\left( {n - 1} \right)}} }}{\mu }} \right\|^{2}\\&\quad = \left\| {divp_{m} - \frac{{2\gamma z^{{\left( {n - 1} \right)}} }}{\mu }} \right\|^{2} + 2\tau \left\langle {div\eta ,\;divp_{m} - \frac{{2\gamma z^{{\left( {n - 1} \right)}} }}{\mu }} \right\rangle + \tau^{2} \left\| {div\eta } \right\|^{2}\\&\quad  \le \left\| {divp_{m} - \frac{{2\gamma z^{{\left( {n - 1} \right)}} }}{\mu }} \right\|^{2} - \tau \left( {2\left\langle {\eta ,\nabla \;\left( {divp_{m} - \frac{{2\gamma z^{{\left( {n - 1} \right)}} }}{\mu }} \right)} \right\rangle - \kappa^{2} \tau \left\| \eta \right\|^{2} } \right)\end{aligned}  $$

Now, consider the following equation

$$ 2\left\langle {\eta ,\nabla \left( {divp_{m} - \frac{{2\gamma z^{{\left( {n - 1} \right)}} }}{\mu }} \right)} \right\rangle - \kappa^{2} \tau \left\| \eta \right\|^{2} = \sum\limits_{i,j \in \Omega } {\left[ {2\eta_{i,j} \cdot \left( {\nabla \left( {divp_{m} - \frac{{2\gamma z^{{\left( {n - 1} \right)}} }}{\mu }} \right)} \right)_{i,j} - \kappa^{2} \tau \left| {\eta_{i,j} } \right|^{2} } \right]} $$

where $\left( {i,j} \right)$ is any point of image region $\Omega$ (2-dimensional matrices).

For every point $\left( {i,j} \right)$, we get

14 $$ \begin{aligned} 2\eta_{i,j} \cdot \left( {\nabla \;\left( {divp_{m} - \frac{{2\gamma z^{{\left( {n - 1} \right)}} }}{\mu }} \right)} \right)_{i,j} - \kappa^{2} \tau \left| {\eta_{i,j} } \right|^{2} \hfill \\ = \left( {1 - \kappa^{2} \tau } \right)\left| {\eta_{i,j} } \right|^{2} + \left| {\left( {\nabla \;\left( {divp_{m} - \frac{{2\gamma z^{{\left( {n - 1} \right)}} }}{\mu }} \right)} \right)_{i,j} } \right|^{2} - \left| {\frac{{\left| {\left( {\nabla \left( {divp_{m} - \frac{{2\gamma z^{{\left( {n - 1} \right)}} }}{\mu }} \right)} \right)_{i,j} } \right|}}{{g_{i,j} }}p_{i,j} } \right|^{2} \hfill \\ \end{aligned} $$

By $\left| {p_{i,j}^{n + 1} } \right| \le g_{i,j} \left( {\mathbf{x}} \right)$, we know

$$ \left| {\frac{{\left| {\left( {\nabla \left( {z^{{\left( {n - 1} \right)}} - \frac{\mu }{2\gamma }\left( {{\text{div}}p_{m} } \right)} \right)} \right)_{i,j} } \right|}}{{g_{i,j} }}\left( {p_{m + 1} } \right)_{i,j} } \right| \le \left| {\left( {\nabla \left( {z^{{\left( {n - 1} \right)}} - \frac{\mu }{2\gamma }\left( {{\text{div}}p_{m} } \right)} \right)} \right)_{i,j} } \right| $$.

So, if $\delta t \le 1/\kappa^{2}$, $divp_{m + 1} - {{2\gamma z^{{\left( {n - 1} \right)}} } \mathord{\left/ {\vphantom {{2\gamma z^{{\left( {n - 1} \right)}} } \mu }} \right. \kern-0pt} \mu }$ is decrease on *n*.

And when $\eta = 0$, it holds $p_{m + 1} = p_{m}$.

In fact, If $\delta t < 1/\kappa^{2}$, it is obvious that $\eta = 0$ is equivalence to $p_{m + 1} = p_{m}$ ;If $\delta t = 1/\kappa^{2}$, by Eq. (14), for any $i,j$ of $\Omega$, it hold

$$ \left| {\left( {\nabla \left( {divp_{m} - \frac{{2\gamma z^{{\left( {n - 1} \right)}} }}{\mu }} \right)} \right)_{i,j} } \right|^{2} = \left| {\frac{{\left| {\left( {\nabla \left( {divp_{m} - \frac{{2\gamma z^{{\left( {n - 1} \right)}} }}{\mu }} \right)} \right)_{i,j} } \right|}}{{g_{i,j} }}\left( {p_{m + 1} } \right)_{i,j} } \right|^{2} , $$

We deduce $\left| {\left( {\nabla \;\left( {divp_{m} - {{2\gamma z^{{\left( {n - 1} \right)}} } \mathord{\left/ {\vphantom {{2\gamma z^{{\left( {n - 1} \right)}} } \mu }} \right. \kern-0pt} \mu }} \right)} \right)_{i,j} } \right| = 0$ or $\left| {\left( {p_{m + 1} } \right)_{i,j} /g_{i,j} } \right| = 1$, In both cases, (12) yields $p_{m + 1} = p_{m}$.

In the following, we will prove the convergence of $\frac{\mu }{2\gamma }divp_{m}$. Let $s = \mathop {\lim }\limits_{m \to \infty } \left\| {divp_{m} - {{2\gamma z^{{\left( {n - 1} \right)}} } \mathord{\left/ {\vphantom {{2\gamma z^{{\left( {n - 1} \right)}} } \mu }} \right. \kern-0pt} \mu }} \right\|$, and $\bar{p}$ be the limit of a converging subsequence $\left\{ {p_{{m_{k} }} } \right\}$ of $\left\{ {p_{m} } \right\}$. Letting $\bar{p}^{'}$ be the limit of $\left\{ {p_{{m_{k} + 1}} } \right\}$, we have

$$ \bar{p}_{i,j}^{{\prime }} = \frac{{\bar{p}_{i,j} + \delta t\left( {\nabla \left( {{\text{div}}\left( {\bar{p}} \right) - \frac{2\gamma }{\mu }z^{{\left( {n - 1} \right)}} } \right)} \right)_{i,j} }}{{1 + \delta t\left( {g\left( {\mathbf{x}} \right)} \right)_{i,j} \left| {\left( {\nabla \left( {{\text{div}}\left( {\bar{p}} \right) - \frac{2\gamma }{\mu }z^{{\left( {n - 1} \right)}} } \right)} \right)_{i,j} } \right|}} $$

and repeating the previous calculations we see

$$ s = \left\| {div\bar{p} - {{2\gamma z^{{\left( {n - 1} \right)}} } \mathord{\left/ {\vphantom {{2\gamma z^{{\left( {n - 1} \right)}} } \mu }} \right. \kern-0pt} \mu }} \right\| = \left\| {div\bar{p}^{'} - {{2\gamma z^{{\left( {n - 1} \right)}} } \mathord{\left/ {\vphantom {{2\gamma z^{{\left( {n - 1} \right)}} } \mu }} \right. \kern-0pt} \mu }} \right\|. $$

It holds $\bar{\eta }_{i,j} = {{\left( {\bar{p}_{i,j}^{{\prime }} - \bar{p}_{i,j} } \right)} \mathord{\left/ {\vphantom {{\left( {\bar{p}_{i,j}^{{\prime }} - \bar{p}_{i,j} } \right)} {\delta t}}} \right. \kern-0pt} {\delta t}} = 0$, for any $i,j$,i.e., $\bar{p}^{'} = \bar{p}$

So we can deduce

$$ \left( {\nabla \left( {z^{{\left( {n - 1} \right)}} - \frac{\mu }{2\gamma }\left( {{\text{div}}\bar{p}} \right)} \right)} \right)_{i,j} + \frac{{\left| {\left( {\nabla \left( {z^{{\left( {n - 1} \right)}} - \frac{\mu }{2\gamma }\left( {{\text{div}}\bar{p}} \right)} \right)} \right)_{i,j} } \right|}}{{g_{i,j} }}\bar{p}_{i,j} = 0 $$

which is the Euler equation for a solution of (8). One can deduce that $\bar{p}$ solves (8) and that $\mu /2\gamma div\bar{p}$ is the projection of $\pi_{\mu K/2\gamma } \left( {z^{{\left( {n - 1} \right)}} } \right)$. Since this projection is unique, we deduce that all the sequence $\mu /2\gamma divp^{n}$ converges to $\pi_{{{{\mu K} \mathord{\left/ {\vphantom {{\mu K} {2\gamma }}} \right. \kern-0pt} {2\gamma }}}} \left( {z^{{\left( {n - 1} \right)}} } \right)$ as $\kappa^{2} \le 8$.

6b is equivalence to solve the nonlinear system

$$ \left( {1 - fe^{ - z} } \right) + 2\gamma \left( {z - w^{\left( n \right)} } \right) = 0 $$

## Numerical experiment

We compare our algorithm on eliminating staircase effect and preserving the detail to SO model, HNW model and classic iteratively reweighted total variation (CWTV). Signal to Noise Ratio (SNR) of the denoising image to the corresponding true image is defined as

$$ {\text{SNR}}\left( {X,\bar{X}} \right) = 10\lg \left( {\frac{{\left\| {\bar{X}} \right\|^{2} }}{{\left\| {X - \bar{X}} \right\|^{2} }}} \right) $$

where $\bar{X}$ is the denoised image and $X$ is the true image. We stop algorithm while attaining maximum SNR. The test images are, “Shape1”, “Shape2”, “Barbara”, “Lena256”, “Cameraman”, “Phantom”. The multiplicative noise with standard variance (NSV) of 1/30 and 1/10 are considered in our experiments. Table 1 shows the effect of artificial parameter *ε*_*n*_ to denoising results of classic iteratively reweighted isotropous total variation method. Table 2 is the comparison of denoising results on SNR. From Table 1, we can explicitly see that suitable artificial parameter *ε*_*n*_ can obtain better denoising results than some other models (such as SO model, HNW model), while unsuitable artificial parameter *ε*_*n*_ obtain lower SNR than other models. New algorithm can obtain the highest SNR than SO model, HNW model and classic iteratively reweighted method. Moreover the new algorithm is not affected by this parameter.

**Table 1:** The effect of artificial parameter *ε*_*n*_ to denoising results (dB)

Test images	NSV	$\varepsilon_{n} = \frac{1}{n + 2}$	$\varepsilon_{n} = \frac{1}{{\left( {n + 2} \right)^{2} }}$	$\varepsilon_{n} = \frac{1}{{2^{n + 1} }}$	$\varepsilon_{n} = \frac{1}{2}$	$\varepsilon_{n} = 0.1$	New algorithm
Shape1	1/30	13.1487	13.2599	13.1100	12.0367	12.5180	15.6686
	1/10	12.3661	11.6466	10.6260	12.2160	12.1057	12.3856
Shape2	1/30	19.4796	18.7226	18.8344	19.2633	18.7180	19.9760
	1/10	15.8457	14.9985	15.6592	15.1059	15.5034	16.0540
Barbara	1/30	12.5857	12.2622	12.0967	12.0016	12.1491	13.0724
	1/10	10.3475	9.4710	9.5138	9.4252	10.0087	10.3138
Lena256	1/30	13.5712	13.2305	13.2450	13.1860	13.0905	14.0905
	1/10	10.5605	10.1580	10.1726	10.1229	10.0934	10.9022
Cameraman	1/30	16.4723	15.1124	16.0535	15.7450	16.1120	16.3157
	1/10	13.1132	11.2307	11.9135	11.8059	13.0282	13.5304
Phantom	1/30	18.7465	17.4713	18.2407	18.0309	18.7136	20.3229
	1/10	15.6020	14.5858	15.0706	15.0785	15.2035	15.6289
Average		14.3199	13.5125	13.7113	13.6682	13.9370	14.9384

**Table 2:** Comparison of denoising results on SNR (dB)

Test images	NSV	SO	HNW	CWTV	New algorithm
Shape1	1/30	10.6622	11.4753	13.2599	15.6686
	1/10	7.9142	9.2177	12.3661	12.3856
Shape2	1/30	16.9584	19.2644	19.4796	19.9760
	1/10	12.9863	15.5438	15.8457	16.0540
Barbara	1/30	11.1623	12.5344	12.5857	13.0724
	1/10	8.5457	10.3088	10.3475	10.3138
Lena256	1/30	12.4623	13.1826	13.5712	14.0905
	1/10	9.6806	10.5069	10.5605	10.9022
Cameraman	1/30	14.8305	15.9641	16.4723	16.3157
	1/10	11.7141	13.5090	13.1132	13.5304
Phantom	1/30	18.5579	19.7853	18.7465	20.3229
	1/10	14.6484	14.7103	15.6020	15.6289
Average		12.5102	13.8336	14.3292	14.9384

### Experiment 1: Comparison on eliminating staircase effect

**Fig. 1:**
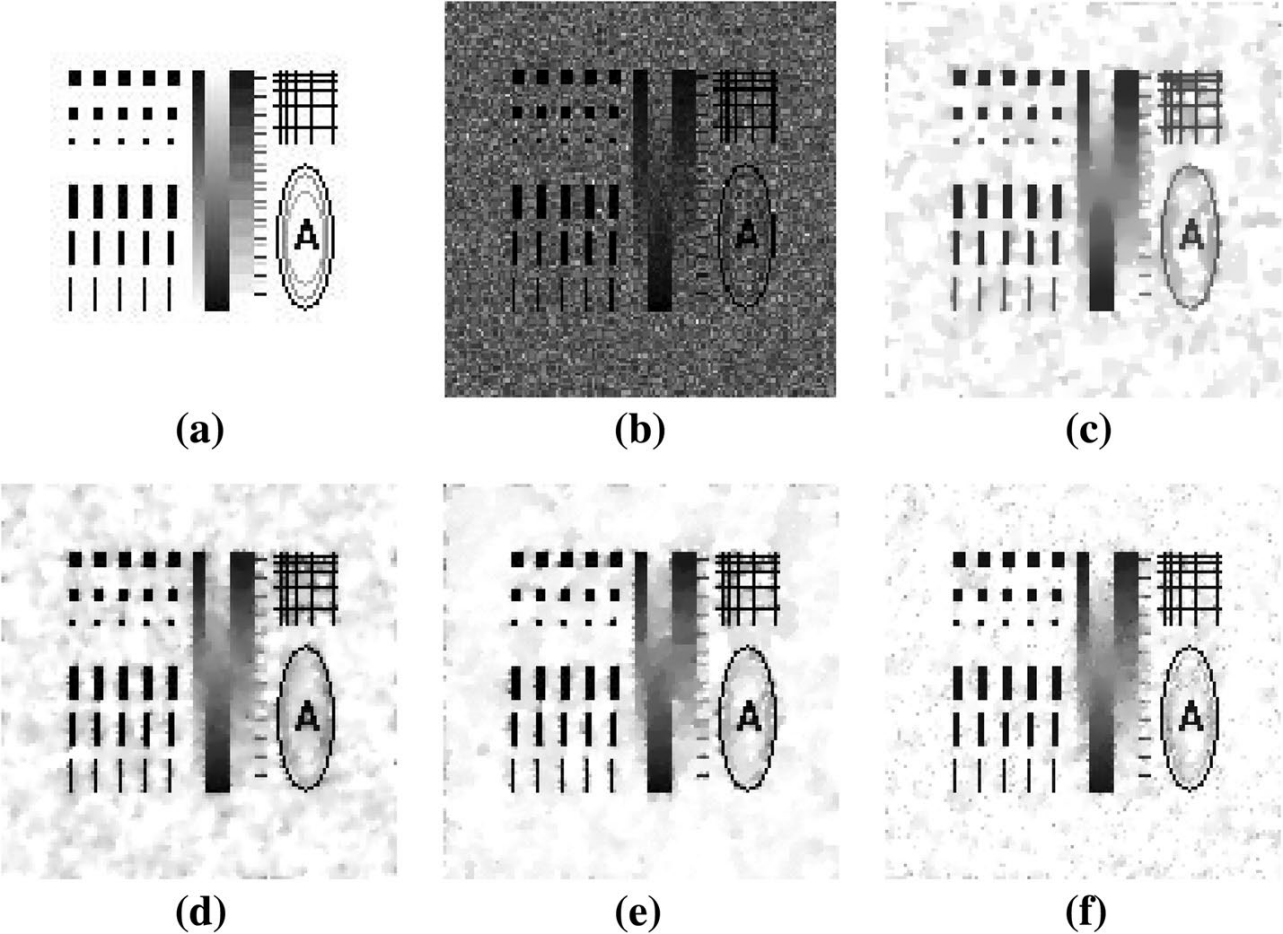
Experimental results on Shape1 image (multiplicative noise with standard variance 1/10). **a** Original image; **b** noisy image; **c** denoised image by SO (SNR = 7.9142 dB); **d** denoised image by HNW (SNR = 9.2177 dB); **e** denoised image by classic iteratively reweighted algorithm (SNR = 12.3661 dB); **f** denoised image by our algorithm (SNR = 12.3856 dB)

“Shape1” is used as a test image in this experiment, the multiplicative noise intensity is standard variance 1/10. In our algorithm, $\mu = 0.013$ and the number of inner iteration is set 30, the denoising SNR result can achieve 12.3856 dB. Figure 1 is the denoising results. Comparing Fig. 1c–f, we can see, staircase effect is restrained in the alternative splitting minimizating algorithm (HNW model and our algorithm), and the transition of smooth region in the new model has a good visual effect. Moreover, we can clearly find new model can preserve edge and detail better than SO model, HNW model. The edge and details of the restored images are preserved because of the action of the weighted function. In Fig. 1 short widthways lines in our methods can be restored more number than SO model and HNW model.

### Experiment 2: Detail preserving

**Fig. 2:**
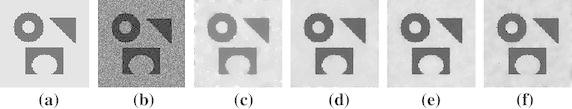
Experimental results on Shape2 image (multiplicative noise with standard variance 1/10). **a** Original image; **b** noisy image; **c** denoised image by SO (SNR = 12.9863 dB); **d** denoised image by HNW (SNR = 15.5438 dB); **e** denoised image by classic iteratively reweighted algorithm (SNR = 15.8457 dB); **f** denoised image by our algorithm (SNR = 16.0540 dB)

**Fig. 3:**
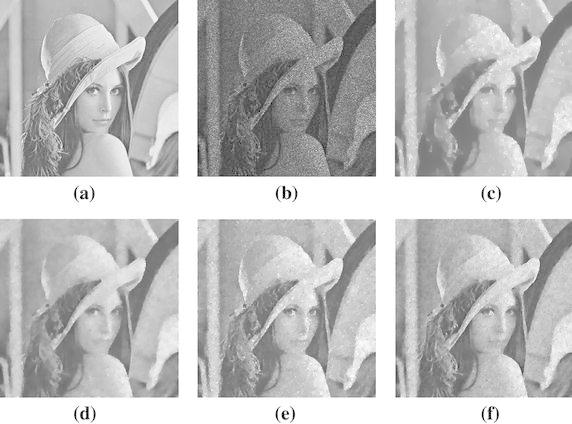
Experimental results on Lena256 image (multiplicative noise with standard variance 1/10). **a** Original image; **b** noisy image; **c** denoised image by SO (SNR = 9.6806 dB); **d** denoised image by HNW (SNR = 10.5069 dB); **e** denoised image by classic iteratively reweighted algorithm (SNR = 10.5605 dB); **f** denoised image by our algorithm (SNR = 13.9022 dB)

“Shape 2” and “Lena256” images are contaminated by multiplicative noise with standard variance 1/10. Figures 2 and 3 are the denoising results. In our algorithm to “Shape 2”, $\mu = 0.015$ and the number of inner iteration is set 30, the denoising SNR result can achieve 16.0540 dB. We can see the denoising results is better than the SO model and HNW model. In our algorithm to “Lena256”, $\mu = 0.0025$ and the number of inner iteration is same as the experiment 1, and the denoising SNR result can achieve 13.9022 dB. The preserved detail of our algorithm is better than the SO model and HNW model, especially the feather on the cap.

On the edge of the image, the derivative of image edges is bigger, then weight function value becomes little and the degree of polishing is weakened to the edges. thus the edges are preserved; On the other hand, The derivative of the smooth regions is much small, weighted function is large, which strengthen the smoothing to relatively smooth regions, thus the noise is removed. Compare to Figs. 2 and 3c–f, it is obvious that the denoising results of proposed algorithm can keep details better.

## Conclusion

We study a new algorithm on iteratively reweighted to remove multiplicative noise model. An alternating minimization method is employed to solve the proposed model. And a Chambolle projection algorithm to iteratively reweighted model is proposed. Our experimental results have shown that the quality of images restored by the proposed method is quite good, especially on preserving the detail and restraining the staircase effect. Moreover the proposed algorithm provides an approach to solve the non-convex problem.
